# Retraction Note: Predictive modelling and identification of key risk factors for stroke using machine learning

**DOI:** 10.1038/s41598-026-47902-y

**Published:** 2026-04-13

**Authors:** Ahmad Hassan, Saima Gulzar Ahmad, Ehsan Ullah Munir, Imtiaz Ali Khan, Naeem Ramzan

**Affiliations:** 1 Department of Computer Science, COMSATS University Islamabad, Wah Campus, Grand Trunk Road, Wah, 47010 Pakistan; 2 Department of Computer Science, Cardiff School of Technologies, Llandaff Campus, Western Avenue, Cardiff, CF5 2YB UK; 3 School of Computing, Engineering and Physical Sciences, University of the West of Scotland, High Street, Paisley, PA1 2BE UK

Retraction of: *Scientific Reports* 10.1038/s41598-024-61665-4, published online 20 May 2024

The Editors have retracted this Article.

The Authors have used a publicly available dataset (The Stroke Prediction Dataset). Concerns have been raised regarding the provenance and validity of this dataset [1]. The Authors have been unable to provide any further information regarding the provenance or accuracy of the data included in this dataset. As this dataset was used to train and assess the machine-learning models examined in this Article, the Editors no longer have confidence in the reliability of the results and conclusions of this Article.

All Authors disagree with the retraction.
